# Lymphocyte subsets in experimental rhinovirus infection in chronic obstructive pulmonary disease^☆^

**DOI:** 10.1016/j.rmed.2013.09.010

**Published:** 2014-01

**Authors:** Patrick Mallia, Simon D. Message, Marco Contoli, Katrina Gray, Aurica Telcian, Vasile Laza-Stanca, Alberto Papi, Luminita A. Stanciu, Sarah Elkin, Onn M. Kon, Malcolm Johnson, Sebastian L. Johnston

**Affiliations:** a Airway Disease Infection Section, National Heart and Lung Institute, Imperial College, Norfolk Place, London W2 1PG, United Kingdom; b Imperial College Healthcare NHS Trust, London, United Kingdom; c Research Centre on Asthma and COPD, University of Ferrara, Ferrara, Italy; d GlaxoSmithKline, Uxbridge, Middlesex UB11 1BT, United Kingdom

**Keywords:** Chronic obstructive pulmonary disease, Acute exacerbations of COPD, Respiratory viruses, T lymphocytes

## Abstract

**Background:**

COPD is associated with increased numbers of T cells in the lungs, particularly CD8+ T cells. The mechanisms of increased T cells are unknown but may be related to repeated virus infections in COPD patients. We analysed lymphocyte subsets in blood and bronchoalveolar lavage in smokers and COPD subjects during experimental rhinovirus infections.

**Methods:**

Lymphocytes were isolated from blood and bronchoalveolar lavage from COPD subjects and non-obstructed smokers prior to, and following experimental rhinovirus infection. Lymphocyte surface markers and intracellular cytokines were analysed using flow cytometry.

**Results:**

Following rhinovirus infection CD4+ and CD8+ T cell numbers in the COPD subjects were significantly reduced in blood and CD3+ and CD8+ T cells increased in bronchoalveolar lavage compared to baseline. T cells did not increase in BAL in the control subjects. CD3+ T cells correlated with virus load.

**Conclusions:**

Following rhinovirus infection T cells move from the circulation to the lung. Repeated virus infections may contribute to T cell accumulation in COPD patients.

## Introduction

Chronic obstructive pulmonary disease (COPD) is a growing global epidemic and its prevalence is expected to increase markedly in the future. COPD is an inflammatory condition predominantly caused by exposure to tobacco smoke and a number of inflammatory cells have been implicated in the development of COPD including neutrophils, macrophages and T lymphocytes. COPD is associated with increased numbers of T cells and CD8+ T cells in lung parenchyma and small airways correlate with disease severity [1–4], suggesting these cells are involved in the development and progression of COPD. CD4+, B, NK and gamma delta (γδ) cells have also been identified in the airways of COPD patients, although their relationship with disease pathogenesis is less well established [5]. The mechanisms whereby T cells are increased in the lungs in COPD remain undetermined. T cells are an important component of the adaptive immune response to virus infections and respiratory virus infections are a common cause of acute exacerbations in COPD patients [6]. It has been proposed that repeated virus infections in COPD patients may contribute to T cell accumulation [7] but this hypothesis remains unproven as some studies report increased lymphocytes in COPD exacerbations [8] whereas others do not [9]. We have previously reported increased lymphocytes in bronchoalveolar lavage in COPD subjects infected with rhinovirus [10]. In the present study we characterised the phenotype of lymphocytes in blood and bronchoalveolar lavage during experimental rhinovirus infection in COPD subjects and non-obstructed smokers. We hypothesized that virus infection is associated with recruitment of circulating T cells to the lung, and that this is one potential mechanism of increased numbers of T cells in COPD.

## Methods

### Study participants

COPD subjects (GOLD stage II) (*N* = 11) and smokers (*N* = 12) with a similar smoking history but with normal lung function were recruited. Ethical approval was obtained from the Local Research Ethics Committee (study number 00/BA/459E) and informed consent obtained from all subjects. All subjects were free from respiratory tract infection for the previous 8 weeks and none had received treatment with oral, inhaled or nasal topical steroids, long-acting β-agonists or tiotropium in the previous 3 months. The clinical, inflammatory and virologic data from the experimental rhinovirus infection study have been reported in a previous publication [10]. Blood and BAL for lymphocyte analysis were collected at baseline prior to inoculation with rhinovirus, and on days 7 and 42 post-inoculation.

### Isolation of lymphocytes

Peripheral blood mononuclear cells (PBMC) were obtained by centrifuging whole blood on a separation gradient (Lymphoprep^®^, Axis Shield). BAL fluid was obtained by instillation of 0.9% saline into a peripheral bronchus via a bronchoscope as previously described [10]. PBMC and BAL cells were washed and adjusted to 2 × 10^6^/mL. Cells for surface staining were kept at 4 °C and cells for measurement of intracellular cytokines incubated with phorbol myristate acetate, ionomycin and brefeldin at 37 °C in 5% CO_2_ for 4 h. Cells were washed twice and 50 μL of cell suspension added to antibody for surface markers and incubated for 45 min. Cells for surface staining were fixed and cells for intracellular staining were resuspended in cytofix cytoperm (Becton Dickinson) for 20 min, washed twice, antibody added for 45 min, washed and resuspended. Flow cytometry analysis of cells was carried out immediately following completion of the staining protocol.

### Flow cytometry

All cells were stained with conjugated antibodies (BD Pharmingen) and analysed on a Fluorescence Activated Cell Sorter (Becton Dickinson). Gain and amplitude settings were consistent throughout the study for each subject. PBMC and BAL cells were analysed for lymphocyte surface marker expression by three- and four-colour flow cytometry as described previously [11,12]. The lymphocyte subsets measured were T cells (CD3+), CD4 T cells (CD3+CD4+CD8-), CD8 T cells (CD3+CD8+CD4−), NK cells (CD3-CD16+ CD56+), γδ T cells (CD3-CD4-CD8-γδ+) and B cells (CD3-CD19+). Sufficient cells for measurement of intracellular cytokines were obtained from blood only. Following cell stimulation the frequency of CD4+ and CD8+ cells staining positively for interferon-gamma (IFN-γ) and interleukin-4 (IL-4) were measured [11]. Analysis was performed on at least 10,000 lymphocyte events using Cellquest and Winlist software.

### Inflammatory mediators and virus load

Interleukin (IL)-6, IL-8 and tumour necrosis factor-alpha (TNF)-α were measured in BAL supernatants using enzyme-linked immunosorbent assays (ELISA) performed according to the manufacturers' instructions. Plates were read on a Spectramax Plus 384 plate reader and the results read using SoftMax Pro software. The sensitivities and sources of the individual ELISAs were as follows: IL-6 (3.9 pg/mL), IL-8 (3.9 pg/mL) (R&D Systems, Abingdon, UK) and TNF-α (5 pg/mL) (Biosource, USA). Serum CRP and peripheral blood cell counts were measured in the Clinical Biochemistry and Haematology laboratories of St Mary's Hospital, Imperial College Healthcare NHS Trust. qPCR was performed on 2 μL of cDNA to detect picornavirus in nasal lavage, an unprocessed plug of induced sputum, and unprocessed BAL, using AmplitaqGold DNA polymerase (PE Biosystems ABI Prism 7700). A standard curve was produced by using serially diluted cloned product and results expressed as copies/mL The sensitivity of this assay was 10^4^ copies/mL Virus load was measured with a real-time quantitative RT-PCR assay [10].

### Statistical analysis

Data are presented as medians with changes from baseline analysed with Friedman test. Correlations between data sets were examined using Spearman's correlation. Differences were considered significant for all statistical tests at *P* values of less than 0.05 and all reported *P* values were two-sided. Analysis was performed using GraphPad Prism version 4.00 for Windows (GraphPad Software, San Diego USA).

## Results

### Clinical outcomes

The symptomatic, physiological and inflammatory changes induced by rhinovirus infection have been described previously [10]. Briefly 11 COPD subjects (GOLD stage II using short-acting bronchodilators only) and 12 smokers with normal lung function were included in the study and their clinical characteristics are shown in Table 1. Infection was confirmed by detection of rhinovirus with PCR in nasal lavage, sputum or BAL. 10 of the COPD subjects developed an exacerbation according to our predetermined symptom criteria.

### Blood lymphocytes

**Figure 1:**
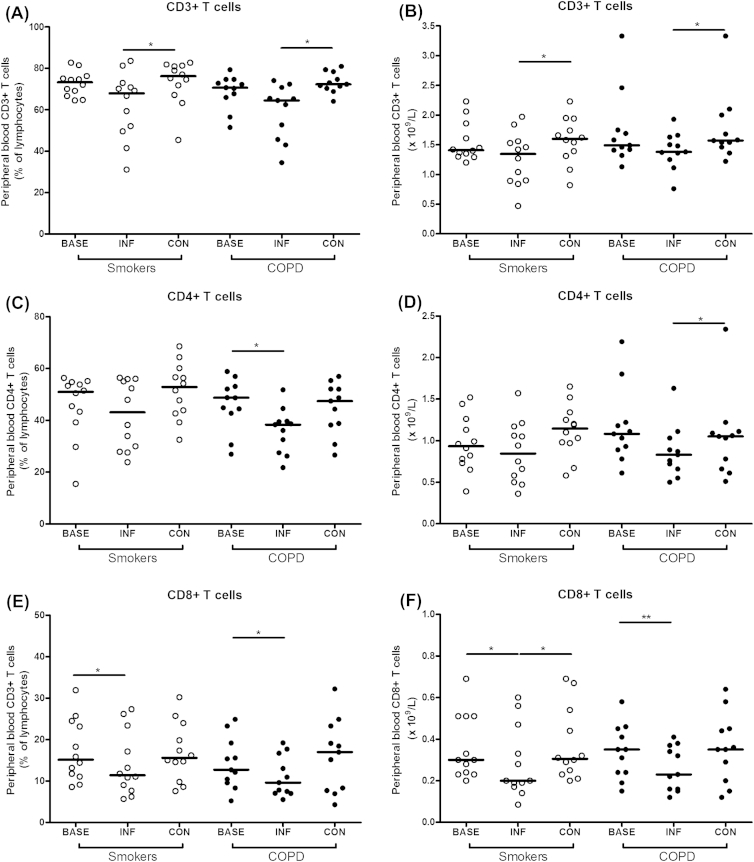
Lymphocyte subsets (medians) in blood at baseline, infection and convalescence in COPD subjects and non-obstructed smokers. Panel A – CD3+ T cell percentages, Panel B – CD3+ T cell numbers, Panel C – CD4+ T cell percentages, Panel D – CD4+ T cell numbers, Panel E − CD8+ T cell percentages, Panel F – CD8+ T cell numbers. *P < 0.05, **P < 0.01.

At baseline prior to infection there were no differences in any of the lymphocyte subsets in blood between the groups, although there was a trend towards higher numbers of NK cells in the COPD group (Table 2). Following rhinovirus infection there was a trend towards reduced in blood CD3+ T cells compared to baseline, but this was not statistically significant. There was a significant increase in CD3+ T cells at convalescence compared to the infection time point (Fig. 1A and B). Blood CD4+ T cells were significantly reduced at infection compared to baseline in the COPD group but not in the smokers (Fig. 1C and D). CD8+ T cell numbers were reduced at infection compared to baseline and convalescence in both groups (Fig. 1E and F). There were no significant changes in blood B cells, NK cells, γδ cells or CD4+/CD8+ ratio following infection in either of the groups (data not shown).

### BAL lymphocytes

**Figure 2:**
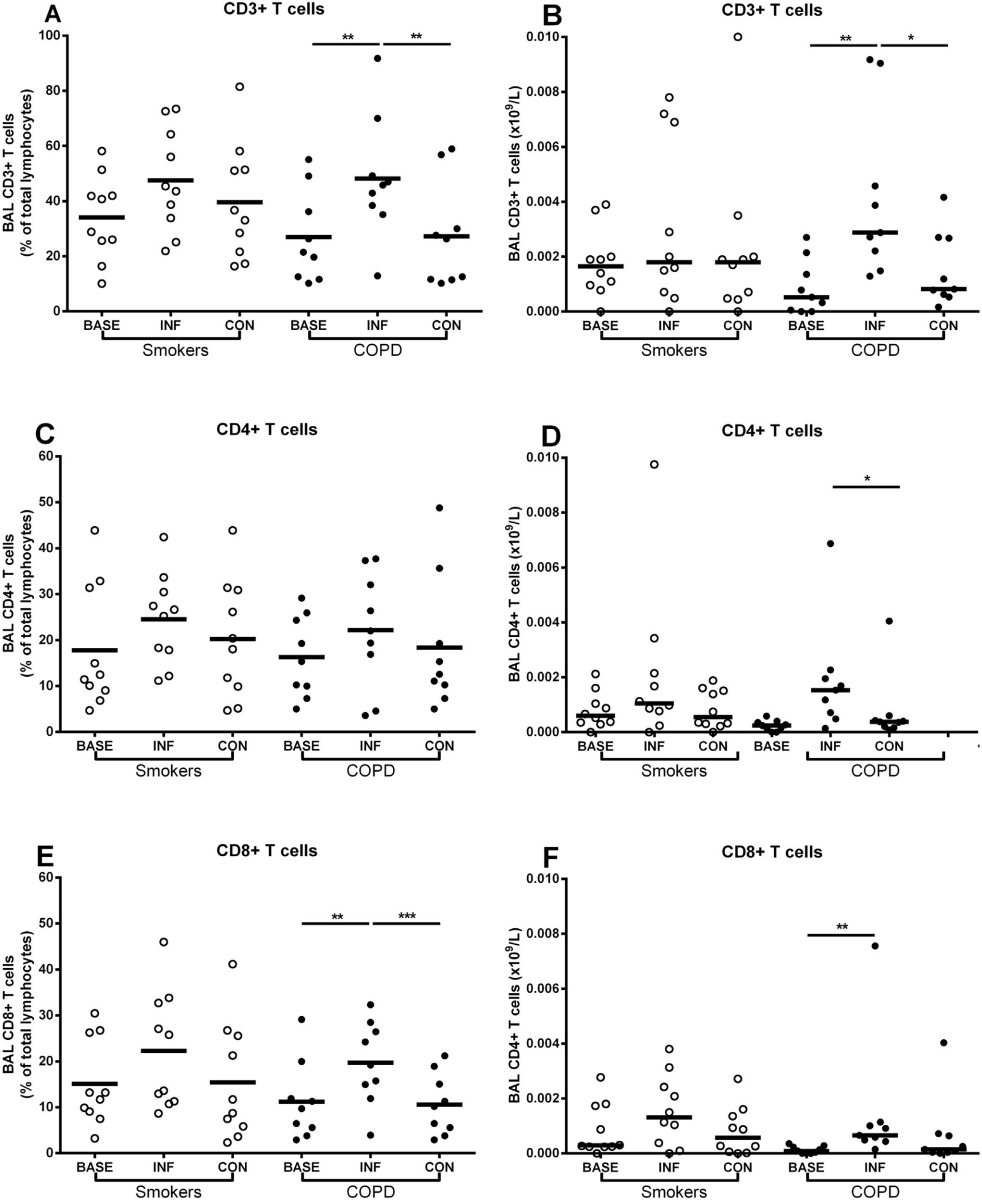
Lymphocyte subsets in BAL at baseline, infection and convalescence in COPD subjects and non-obstructed smokers. Panel A – CD3+ T cell percentages, Panel B – CD3+ T cell numbers, Panel C – CD4+ T cell percentages, Panel D – CD4+ T cell numbers, Panel E − CD8+ T cell percentages, Panel F – CD8+ T cell numbers. *P < 0.05, **P < 0.01.

Sufficient BAL for analysis of lymphocytes was obtained from 9 COPD subjects and 10 smokers. There were no differences in any of the lymphocyte subsets at baseline between the groups (data not shown). Following rhinovirus infection there were significant increases in both percentages and numbers of CD3+ and CD8+ T cells in BAL in the COPD group, but no significant changes in the smokers (Fig. 2). CD4+ T cell numbers were significantly higher at infection compared to convalescence in the COPD group but there were no significant differences in the smokers.

### T cell intracellular cytokine expression

At baseline there were no differences between groups in CD4+ IFN-γ+, CD4+ IL-4+, CD8+ IFN-γ+ or CD8+ IL-4+ T cells in blood (data not shown). There were no significant changes in any of these lymphocyte subsets after infection in either the COPD or the control groups. We also analysed CD4+ IFN-γ+/CD4+ IL-4+ and CD8+ IFN-γ/CD8+ IL-4+ ratios and found no differences between groups or between the baseline and infection time points (data not shown).

### Relationships between BAL and blood lymphocytes and clinical parameters

**Figure 3:**
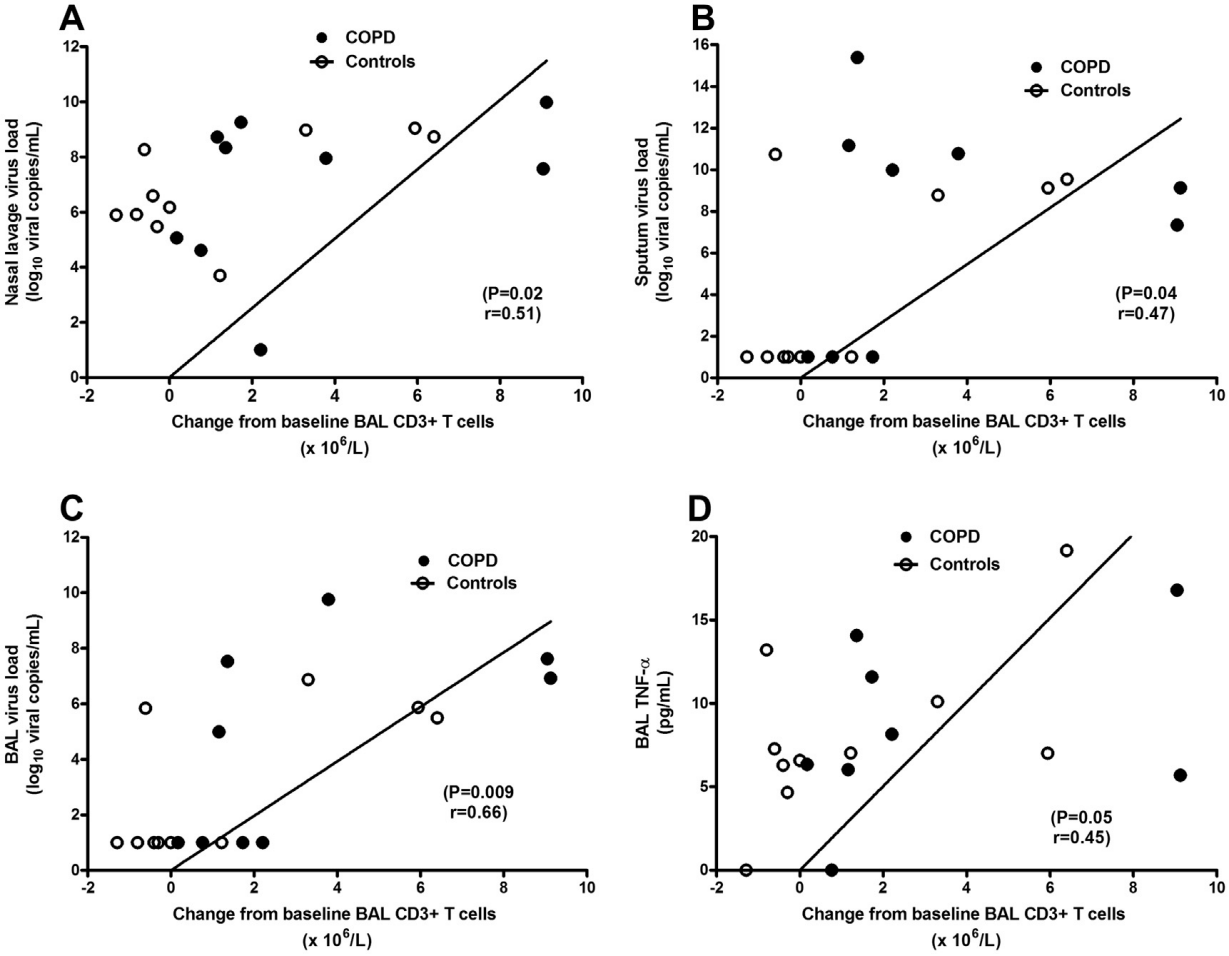
Correlations between CD3+ T cells in bronchoalveolar lavage and virus load in nasal lavage (Panel A), induced sputum (Panel B) and bronchoalveolar lavage (Panel C). Panel D shows the relationship between CD3+ T cells and levels of TNF-α in bronchoalveolar lavage.

In the study population as a whole blood CD3+ T cells at infection correlated inversely with peak serum C-reactive protein (CRP) (*P* = 0.045, *r* = −0.42), peak blood total leukocytes (*P* = 0.047, *r* = −0.42). The relationship between CD3+ T cells and virus load is shown in Fig. 3. The change from baseline in BAL CD3+ T cells at infection correlated with virus load in nasal lavage (Panel A), induced sputum (Panel B) and BAL (Panel C) (Fig. 3). There was a trend towards a correlation between CD3+ T cells in BAL and levels of TNF-α in BAL at infection (Panel D).

## Discussion

This is the first study to prospectively study lymphocyte subsets in treatment-naive virus-induced COPD exacerbations. We report that CD8+ T cells are reduced in blood and increased in BAL in COPD subjects following rhinovirus infection, and that CD3+ T cells in BAL correlate with virus load.

T lymphocytes are increased in stable COPD but the molecular mechanisms by which they accumulate in the airways are not established. There are a number of potential mechanisms including increased recruitment of circulating lymphocytes [13], local proliferation [14] and decreased removal through impaired clearance by macrophages or reduced apoptosis [15]. However both increased [16] and reduced [17] apoptosis of airway lymphocytes in COPD has been reported. T lymphocytes are an essential part of the adaptive immune response to viral infections and therefore their recruitment into the airways will be promoted by respiratory virus infections in COPD patients. However although some studies have reported increased lymphocytes in COPD exacerbations [8,18–20], others have not [6,21–23], so the role of respiratory infections in lymphocyte recruitment has not been established. Increased CD8+ lymphocytes and reduced CD4+/CD8+ ratios in sputum in COPD exacerbations have been reported [9,24]. However in these studies lymphocyte populations at exacerbation were compared with samples collected after treatment with corticosteroids and as corticosteroids are known to increase lymphocyte apoptosis this is likely to have had a major influence on the results [25]. The conflicting results from these studies are likely due to a number of factors including differences in exacerbation aetiology and the effects of treatment, and are difficult to resolve in studies of naturally-occurring exacerbations. We have developed a model of COPD exacerbation using experimental rhinovirus infection that avoids many of the sources of variability inherent in studies of naturally-occurring exacerbations. Using this model we have previously reported increased lymphocytes in BAL following rhinovirus infection [10], and following on from this observation we analysed lymphocyte subsets in blood and BAL in experimental rhinovirus infection.

Following rhinovirus infection in COPD subjects CD4+ and CD8+ T cells were reduced in blood compared to baseline. In BAL CD3+ T cells were increased following infection and this appeared to consist predominantly of CD8+ T cells, as CD4+ T cells in BAL were not significantly increased. Although there were some changes in circulating T cells in the controls, there were no significant increases in BAL following infection in the non-COPD subjects. Therefore this is the first direct evidence that circulating T cells are recruited to the lung in response to virus infection in COPD. Whether this represents an appropriate response required for effective viral clearance, or whether the T cell influx is exaggerated in COPD and results in immune mediated tissue injury remains to be determined. We demonstrated a clear relationship between CD3+ T cells in BAL and virus load in the airways and this was strongest for virus load in BAL. This supports the hypothesis T cells are recruited in response to viral replication in the airways. In our previous study we demonstrated higher virus loads and impaired interferon responses in the COPD subjects [10]. Therefore impaired antiviral innate immunity in COPD may result in higher virus load in the airways and this in turn may drive greater recruitment of T cells. At 6 weeks post-infection T cell numbers had returned to baseline levels and therefore there was no evidence for a persistence of T cells in the airways following virus infection, suggesting that T cell clearance is not impaired in COPD. However in patients with more severe COPD in whom exacerbations are more frequent, repeated viral infections in combination with other mechanisms such as impaired macrophage phagocyotsis [26] and autoimmunity [5] may lead to T cell accumulation in the airways. Further carefully designed studies in patients with more severe COPD will be required to investigate these hypotheses.

NK cells, B cells and γδ cells did not change significantly in blood following rhinovirus infection, however there was insufficient BAL to examine these lymphocytes therefore we cannot conclusively exclude that they are recruited to the airways following virus infection. Regarding the role of Th1/Th2 T cells we did not find any changes in the ratio of IFN-γ/IL-4 CD4+ or CD8+ T cells. Tsoumakidou [9] and Makris [24] reported evidence of a Tc2 profile in COPD exacerbations but their studies differed from ours in that they used sputum rather than BAL, the exacerbations were severe and patients received corticosteroids.

Our study has a number of limitations as it was carried out in a relatively small number of subjects and experimental rhinovirus infection is limited to patients with mild-moderate COPD. Experimental rhinovirus infections in COPD will always be limited by these factors but such studies can provide novel data that is difficult to obtain with naturally-occurring exacerbations and should stimulate further studies in patients with more severe COPD investigating the role of viral infections in T cell recruitment.

In conclusion our study provides evidence that rhinovirus infection is associated with recruitment of circulating T lymphocytes to the lungs in COPD patients and T cell numbers in BAL correlate with virus load. Therefore respiratory virus infections are likely to contribute to T cell recruitment in COPD but clearance of T cells was not impaired. Further studies that include patients with more severe COPD are needed to identify the factors that lead to T cell persistence in the airways in COPD. Defining these mechanisms has the potential to lead to new therapies that inhibit T cell recruitment and may prevent disease progression in COPD.

## Conflict of interests

The authors have no conflict of interests relevant to this manuscript.

## Figures and Tables

**Table 1:** Clinical characteristics of study subjects successfully infected with rhinovirus.

	COPD (*N* = 11)	Controls (*N* = 12)	*P* value
Age (years)	59.6 (47–70)	48.5 (40–58)	*P* = 0.0021
Sex (M/F)	6/5	6/6	*P* = NS
Smoking history (pack-years)	48 (20–109)	34.8 (20–60)	*P* = NS
Current smokers (no.)	8	9	*P* = NS
FEV_1_ (litres)	1.94 (1.23–2.7)	3.58 (2.8–4.76)	*P* < 0.0001
FEV_1_ (% of predicted normal value)	69.73 (62–78)	109.5 (90–128)	*P* < 0.0001
FEV_1_/FVC (%)	55.55 (39–69)	80.33 (73–86)	*P* < 0.0001

**Table 2:** Lymphocyte subsets in blood at baseline in the COPD and the control subjects. All values median and IQR. NK cells – natural killer cells, γδ cells – gamma-delta cells.

	COPD (*N* = 11)	Controls (*N* = 12)	*P* value
CD3+ T cells (×10^9^/L)	1.49 (1.41–1.75)	1.41 (1.31–1.80)	NS
CD4+ T cells (×10^9^/L)	1.08 (0.89–1.22)	0.85 (0.52–1.14)	NS
CD8+ T cells (×10^9^/L)	0.35 (0.24–0.45)	0.30 (0.23–0.51)	NS
B cells (×10^9^/L)	0.178 (0.097–0.441)	0.178 (0.113–0.238)	NS
NK cells (×10^9^/L)	0.118 (0.056–0.175)	0.047 (0.024–0.103)	*P* = 0.07
γδ cells (×10^9^/L)	0.045 (0.015–0.1)	0.0275 (0.011–0.057)	NS
